# Method Development for the Determination of Diallyldimethylammonium Chloride at Trace Levels by Epoxidation Process

**DOI:** 10.1007/s11270-013-1638-6

**Published:** 2013-08-28

**Authors:** Isaac W. Mwangi, J. Catherine Ngila, Patrick Ndungu, Titus A. M. Msagati

**Affiliations:** 1 Department of Applied Chemistry, University of Johannesburg, Doornfontein Campus, P.O. Box 17011, Doornfontein, Johannesburg 2028 South Africa; 2 School of Chemistry, University of KwaZulu-Natal, P/Bag X45001, Westville Durban, 4000 South Africa; 3 Chemistry Department, Kenyatta University, P.O. Box 43844-00100, Nairobi, Kenya

**Keywords:** Polydiallyldimethylammonium chloride, Epoxide, Polycation, UV–Vis spectrophotometry, Treated water

## Abstract

Domestic water is abstracted from its sources in raw form with a high content of dissolved and suspended material. Polydiallyldimethylammonium chloride (polyDADMAC) is a cationic polyelectrolyte used in the initial water clarification process. However, its residues in treated water pose a health risk as they react with chlorine to produce a carcinogenic compound. There is a need to determine the concentration of the polyelectrolyte cations that pass through the flocculation stage before the chlorine disinfection process in water treatment plants to ascertain the safety of water to consumers. The cationic polymer is UV inactive, and previously available methods for determining the concentrations of polyelectrolytes are unsatisfactory due to poor detection limits. This paper describes a UV–Visible (UV–vis) spectrophotometry method for the determination of residual polyDADMAC as an epoxide. The novelty method lies on the epoxidation of polyDADMAC using 20 % sodium hydroxide dissolved in 30 % hydrogen peroxide to produce a UV–Vis active compound. The epoxidation was confirmed by UV–Vis, FTIR and ^1^H NMR techniques. Dilute solutions of varying concentrations of polyDADMAC (0.2–1.0 mg L^−1^) were treated with a basic solution of hydrogen peroxide then analysed by UV–Vis spectrophotometry. The wavelength at maximum absorption (λ_max_) was found to be 313 nm, and a linear calibration curve with a correlation coefficient (*R* ^2^) of 0.993 was used for quantification purposes. The detection limit measured as three times the signal of the blank and was found to be 2.1 × 10^−4^ mg L^−1^. The method was applied to determine the concentration of polyDADMAC spiked in water samples collected from a pool as a model for environmental matrix. The results obtained agreed with the quantities spiked in the solution, thus qualified the method to be suitable for the determination of polyDADMAC in treated waters at trace levels. The method was also used to investigate the adsorption capacity of polyDADMAC on sand filters. The adsorption method was found to be in accordance with Langmuir with an adsorption capacity of 2.068 mg g^−1^.

## Introduction

Polydiallyldimethylammonium chloride (polyDADMAC) is a cationic liquid used as a flocculant in water treatment plants. Its flocculation property is due to high charge density which promotes the agglomeration of suspended particles making it very effective in flocculating, decolouring, killing algae and removing organics such as humus (Sang-kyu and John 1999). It has been widely used in the treatment of drinking and wastewater due to its advantages over inorganic coagulants such as alum and iron salts (Jackson 1981). Particles of all sizes and from different origins including colloids are present in all virtually raw waters, and they are a major cause of objectionable turbidity, colour, taste and odour in drinking water (Jackson 1981). Due to the size of some particles suspended in water, they are impossible to settle and thus would require coagulation. The application of chemical coagulant is done to aid in the clarification to eliminate colloidal particles. Coagulation is a chemical and physical process wherein collisions between colloidal particles and coagulant chemicals result in their cohesion and eventual sedimentation as agglomerates. The mechanism involves neutralization of negative charges on the colloidal particles' surface resulting to the attraction of the now neutral particles by the coagulant to produce spongy masses called flocs (Majam et al. 2004).

In South African water treatment processes, about 75 % of flocculants used are polyDADMAC and epichlorohydrin-dimethylamine (epi-DMA) (Majam et al. 2004). The use of such large quantities of polyelectrolytes results in residues of the unreacted polyelectrolytes in the treated water. The polycation, polyDADMAC, reacts with chlorine to form *N*-nitrosodimethylamine (NDMA) (Choi and Valetine 2003; Hyuckpark and Valetine 2009). The product of this reaction is a human carcinogen and has been found to occur in drinking water (Loeppky and Micheljda 1994). Due to its toxicity (through the formation of NDMA), there is a need to determine the levels of the polycation, with the intention to remove all dissolved polycation after the clarification process and before chlorine disinfectation process for the safety of consumers.

However, there is a challenge in detecting the residual amounts of the coagulants in the final treated waters owing to their weak UV absorbing chromophores (John et al. 2002). The current methods used for measuring residual organic polyelectrolytes in potable water such as potentiometric and colloidal titration are inadequate, making them unreliable for monitoring polyDADMAC in drinking water at trace levels due to poor detection limits, time consuming and lack of reproducibility (Sakai 2001). To overcome the challenge, this study considered the possibility of introducing groups that are UV–Vis sensitive within the chemical structure of polyDADMAC and then analyse the product by UV–Vis spectroscopy. The aim of the study was to develop a method to quantify the polycation at trace levels using UV–Vis method. This was done by forming an epoxide upon treating the polycation with strongly basic solution of hydrogen peroxide.

## Material and Methods

### Chemicals and Reagents

All the solutions were prepared in double distilled water, and all the reagents used were of analytical grade. The polycation, diallyldimethylammonium chloride 65 %, hydrogen peroxide 50 %, methanol magnesium sulphate and sodium hydroxide pellets were all supplied by Sigma Aldrich (St. Louis, Missouri USA).

### Instrumentation

The diallyldimethylammonium chloride and its epoxidised form were characterised using a Shimadzu UV-2450 UV–Vis spectrophotometer (Tokyo, Japan). Characterization of the functional groups was carried out using a Fourier transform infrared (FTIR) spectrophotometer (Perkin Elmer 100 with sampling accessory, Waltham, MA, USA) using the attenuated total reflectance (ATR) mode (Mukamel 2000 ). The cationic polyelectrolyte and its corresponding epoxide were also characterized by means of a 1H NMR, Bruker 400 MZ spectrometer (Hanau, Germany). The spectra were recorded on a Bruker AM-400 spectrometer using TMS as an internal standard (Asakura et al. 2001). Dilute solutions of the polycation were evaporated using a Büchi rotary evaporator model R-210 with a heating bath model B491 (Basel, Switzerland).

### Epoxidation Procedure

**Scheme 1:**
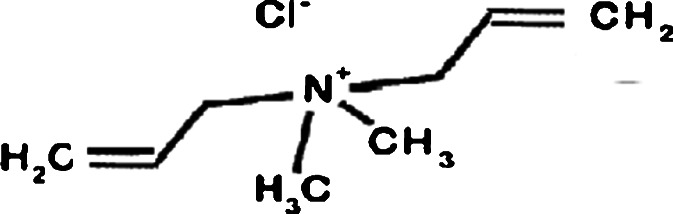
Chemical structure of diallyldimethylammonium chloride

The experiment was done by exploiting the ability of double bonds within the structure of the polycation to be epoxidised with a strongly basic solution of hydrogen peroxide. The structure is shown in Scheme 1.

**Scheme 2:**
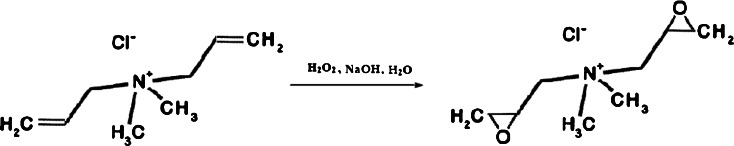
The epoxidation of an electron-deficient double bond

The double alkenyl bonds were epoxidised by treating alkenes with peroxide-containing reagents catalysed by a strong basic environment. The study used alkali-catalysed hydrogen peroxide in situ for the process as described by Bortolini et al. (2002). Scheme 2 shows the epoxidation process.

The mole reacting reactant-product ratio is one to one; thus, the concentration of the epoxide is proportional to the concentration of the reacting arene functional group in the analyte (Díez et al. 2008). A standard epoxidation procedure as described by Bortolini et al. (2002) was adopted. Standard solutions of the unsaturated compound, polyDADMAC, (25 mL) were reacted with 30 mL of 30 % hydrogen peroxide. The reaction mixture was made strongly basic by addition of 25 mL of 20 % sodium hydroxide. The mixture was placed in a water bath at 60 °C with vigorous stirring for 1 h. To the water sample, algae and all the suspended particles were removed from the water sample by filtration using a sintered glass crucible no. 3. This was followed by spiking varying concentrations of polyDADMAC to 1 L of water sample. These solutions were then preconcentrated by evaporation of water through a rotary evaporator. A similar epoxidation procedure done to the model solutions was performed on the sample solutions, and the product was analysed by UV–Vis spectrophotometer to determine the extent of the epoxide in each case.

This method was done by exploiting the epoxidation ability of basic catalysed hydrogen peroxide on the alkenyl double bond of the polycation. The method was then applied to determine the concentration of DADMAC in dilute solutions as well as its adsorption capacity on sand.

### Batch Sorption Experiments

Sorption studies were carried out on a Lab-line mechanical reciprocating shaker model SSL2 (Harrogate, UK) using 100-mL plastic screw cap bottles. Model solutions containing a known concentration of polyDADMAC were prepared, and known weights of the adsorbent material (sand) were added to each of the mixtures and then left to equilibrate for 1 h allowing sufficient time for adsorption. The resulting mixture filtered through Whatman no. 42 filter paper, and the cations in the filtrate were determined by UV–Vis spectroscopy.

## Results and Discussions

### Reaction Mechanism

**Scheme 3:**
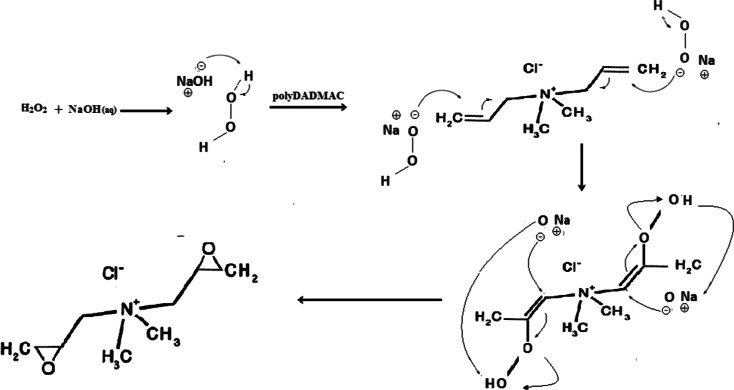
The proposed generic mechanism for nucleophilic epoxidation of an electron-deficient olefins

The proposed reaction mechanism for the epoxidation procedure as described by Díez et al. (2008) on epoxidation of electron deficient alkenyl compounds using alkaline H_2_O_2_ is shown in Scheme 3.

### UV–Vis Characterization

**Fig. 1:**
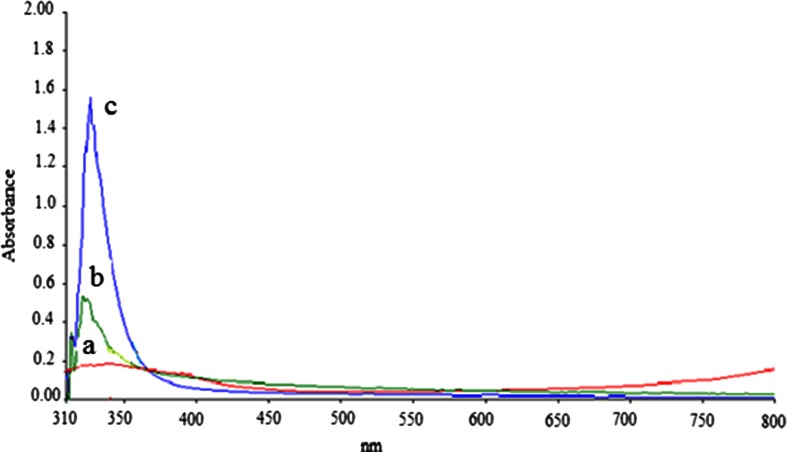
UV–Vis spectra of (*a*) unreacted DADMAC and (*b*) 0.5 mg L^−1^ and (*c*) 2.0 mg L^−1^ epoxidated polyDADMAC

The extent of epoxidation on the polyDADMAC solution treated with hydrogen peroxide under a strong basic environment was studied by UV–Vis spectrophotometry. Two sample solutions with different cation concentrations were used, and the resulting absorption spectrum recorded is presented in Fig. 1.

**Fig. 2:**
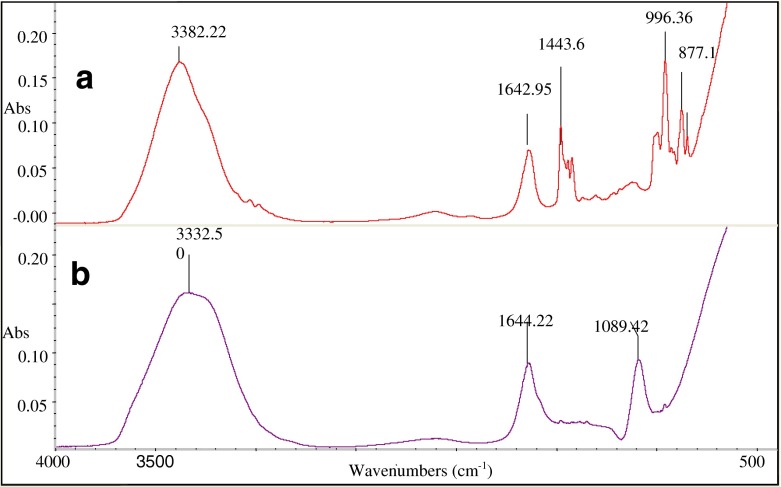
FTIR spectra of DADMAC solution (**a**) and its epoxidated form (**b**)

DADMAC is not UV–Vis active, but after the epoxidation reaction, a sharp band was observed at 313 nm which confirms a successful epoxidation of the double bonds in the polycation rendering it as UV active. A similar peak was observed by Waterfall and Sims (1972) who attributed it to epoxy derivatives. The results show that absorbance at that wavelength is concentration-dependent as demonstrated in spectra in Fig. 2a, b.

### FTIR Characterization

Samples of the polyDADMAC cationic electrolyte and the epoxidised solutions were analysed by ATR-FTIR spectroscopy; the resulting spectra are shown in Fig. 2.

**Fig. 3:**
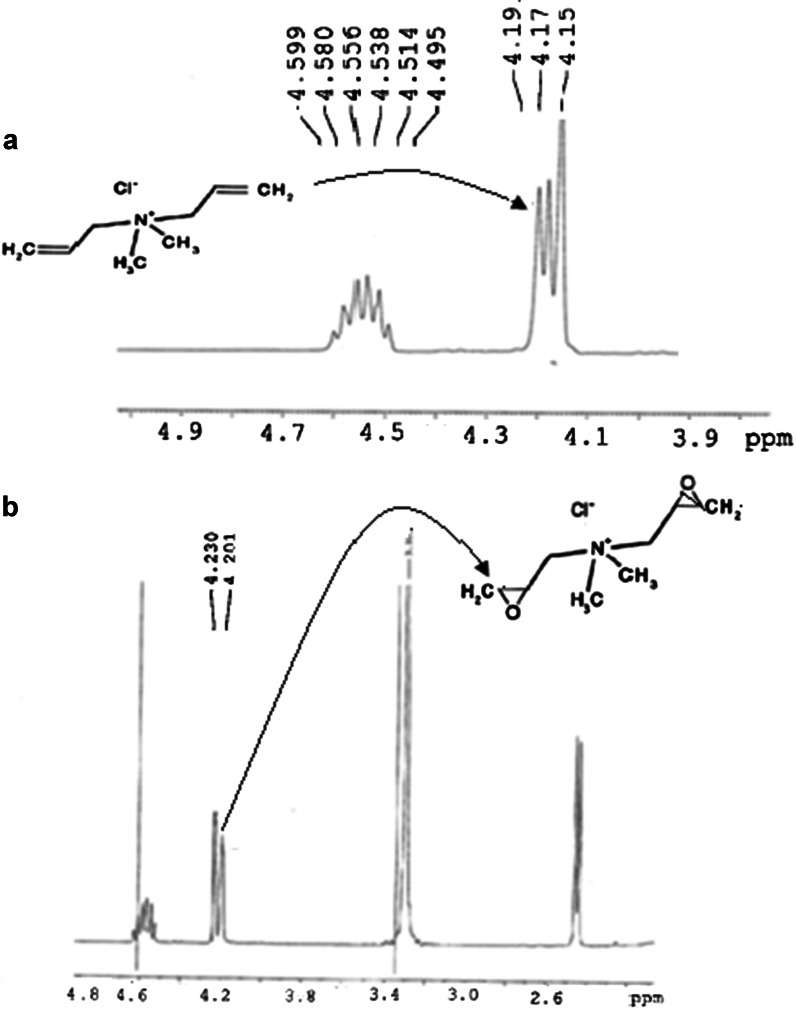
^1^H NMR spectra of DADMAC (**a**) and its epoxidated form (**b**)

The results from FTIR characterization indicated the presence of functional groups found in the materials before and after the epoxidised process. The broad signals at 3,382.22 and 3,332.50 cm^−1^ in spectra A and B, respectively, were assigned to the –OH group contributed by the water which is the solvent for these polycations. The peaks observed at 1,642.95 and 1,644.22 cm^−1^ in the spectra in Fig. 3a, b, respectively, were assigned to C–N stretching vibration as reported by (Vikman and Sipi 2003) who worked with electrophotographic print ink dispersed in 2-pyrrolidone which is a tertiary amine. The signal at 1,443.6 cm^−1^ in Fig. 3a was assigned to C=C stretching, in a positively charged environment contributed by the positively charged nitrogen of the polycations (Vikman and Sipi 2003). The epoxidation process gave rise to a distinct characteristic peak of the epoxy ring at 1,089.42 cm^−1^ as it appears in Fig. 3b (Malutan et al. 2008). The FTIR spectra of polyDADMAC and the epoxyDADMAC in Fig. 3a, b show clear structural differences between the two compounds. This proved that the analysis was a suitable method for the distinction of the overlaid spectra of DADMAC and its epoxidated form.

### ^1^H NMR Characterization

The polycation and its epoxide were characterised by ^1^H NMR, and the results are depicted in Fig. 3.

The ^1^H NMR spectra of the epoxide product (b) has a doublet at 4.20 and 4.23 δ clearly indicating the presence of the proton environments attached to the oxygen bonded to the carbon atom of the epoxy ring (Han et al. 2006). A triplet signal was observed at 4.15, 4.17 and 4.19 δ, which were assigned to the hydrogen atoms of the alkenyl group of polyDADMAC (Han et al. 2006).

### Determination of PolyDADMAC as Epoxide

**Fig. 4:**
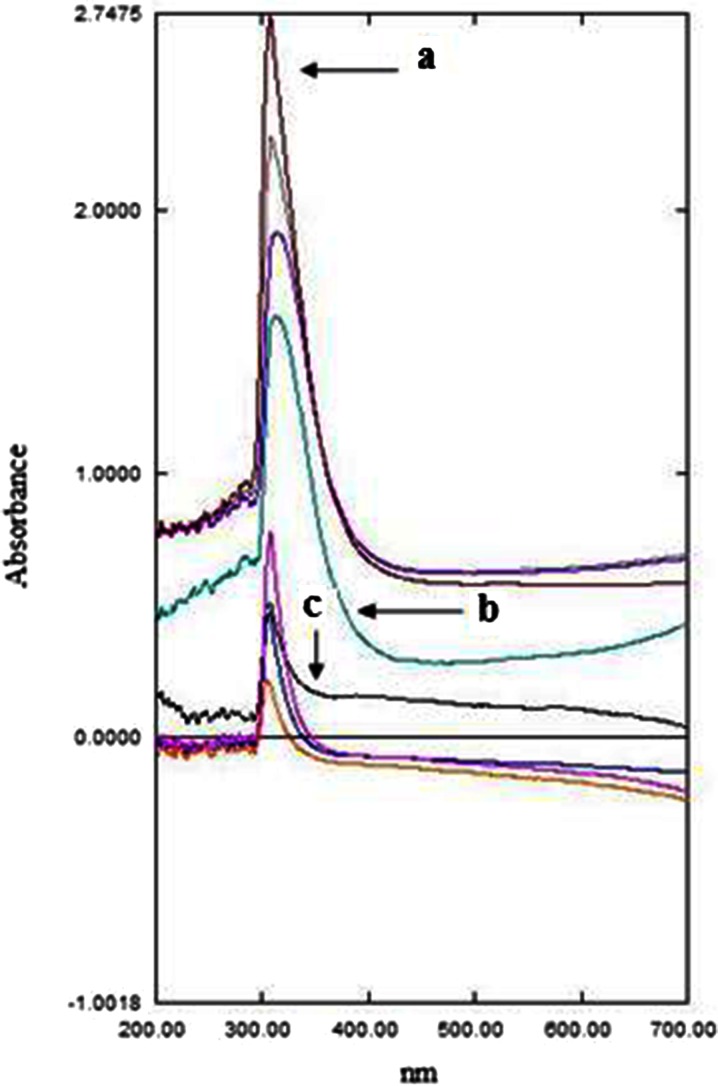
UV–Vis overlaid spectra of various epoxidated polyDADMAC solutions (**a**) 1.5 mg L^−1^, (**b**) 0.8 mg L^−1^ and (**c**) 0.4 mg L^−1^

Different solutions with varying polyDADMAC concentrations were separately epoxidated, and the respective solutions were analysed by UV–Vis spectrometry. The overlaid spectra are shown in Fig. 4.

**Fig. 5:**
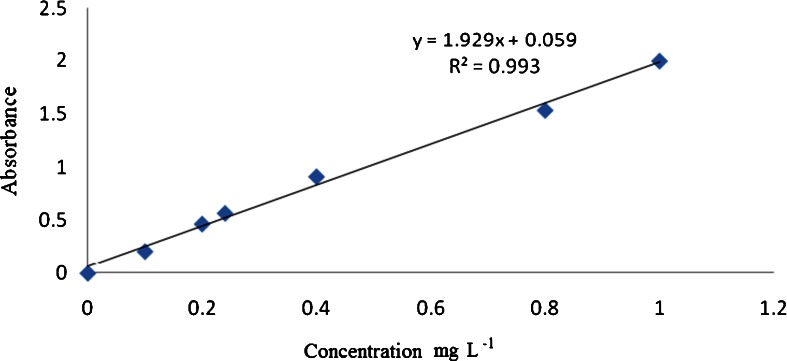
Calibration curve for determination of DADMAC in aqueous media

Results in Fig. 4 show that the instrument response (absorbance) had a direct relationship with the increase in the concentration of the various solutions thus obeyed the Beer–Lambert law. From that data of the different concentrations of the epoxidation procedure, a calibration curve for the determination of DADMAC was obtained and is presented in Fig. 5.

Results (Fig. 5) show the existence of a linear relationship between absorbance and concentration of the epoxidated cationic material. The curve was linear with a correlation coefficient (*R* ^2^) of 0.993. The curve was then applied to evaluate the concentration of DADMAC in unknown solutions.

### Detection Limit

To evaluate the detection limit, the equation $D.L=\overset{-}{x_B}+3{\sigma}_B$ was applied, where $\overset{-}{x_B}$ is the mean of the blank reading, and σ_B_ is the standard deviation. Six blank solutions were made, and their measurements recorded; the mean absorbance of 0.0007 was obtained. When applying the regression equation, this translated to a concentration of 5.76 × 10^−4^ μg L^−1^. The standard deviation was found to be 3.7 × 10^−8^. The limit of detection was found to be 2.1 × 10^−4^ μg L^−1^ indicating that the method is suitable for the determination of polyDADMAC at trace levels.

### Analysis of Water Samples

Water samples were collected from a water pond within the University of Johannesburg, Doornfontein Campus, in the month of March 2012 and were analysed using the above method. As algae had grown in the pond water due to eutrophication, the water samples were filtered using a sintered glass pore size no. 3 before the analysis. Replicate samples were taken, and different known concentrations of polyDADMAC spiked into various 1-L volumes of the sample water were used to make respective analyte solutions. The solutions were preconcentrated by evaporation of water to obtain a final volume of ~ <100 mL. The mixtures were treated in a similar procedure as the model solutions, and the results obtained were recorded in Table 1.

**Table 1:** Concentration of DADMAC spiked in water samples

Sample spiked	Spiked polyDADMAC (mg L^−1^)	Recovered polyDADMAC (mg L^−1^)
1S	0.01	0.012 ± 0.0001
2S	0.05	0.047 ± 0.001
3S	0.1	0.106 ± 0.001
4S	0.5	0.512 ± 0.002
5S	0.8	0.796 ± 0.003
6S	1.0	0.993 ± 0.002
7S	1.2	1.196 ± 0.001
8S	2.0	2.11 ± 0.003

*S* sample replicates

The results of the content of DADMAC obtained by this method agree with the quantities of polyelectrolyte spiked in the solution. Despite the water having an environmental matrix, the results qualify the developed method to be suitable for the determination of polyDADMAC in water at trace levels.

### Effect of Concentration on the Removal of DADMAC by Sand

**Fig. 6:**
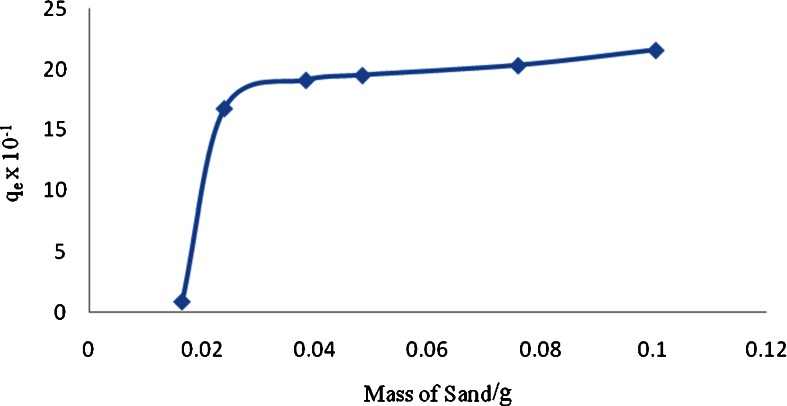
Effect of sorbent dose on adsorption of DADMAC

A normal practice for the removal of unreacted polyDADMAC by water treatment plants is done by adsorption of the polyelectrolyte by sand filters (Nozaic et al. 2000). An experiment was carried out to find out the sorption capacity of sand on this cationic polyelectrolyte using the developed method. Sorption of the DADMAC under study was carried out in batch equilibrium experiments. Batches of 20-mL model solutions (5 μg L^−1^) were prepared and dispensed into 100-mL polyethylene bottles containing approximately varying amounts of sand. The mixtures were equilibrated for 1 h after which the amount of DAMAC remaining in solution determined through the epoxidation process. The results obtained were presented as DAMAC concentration adsorbed in milligram per gram of the sorbent (*q*_e_) against mass of sand sorbent as shown in Fig. 6.

The general observation made was that the adsorption increased with an increase in the mass of sand. A plateau appears after all the binding sites are occupied, where there was little or no significant extra adsorption. This was attributed by the fact that concentration is the driving force for the polycations to occupy available sorption sites on the adsorbent (Ilhan et al. 2004).When most of the available sites on the sorbent are occupied, then this results in a saturation effect showing a steady state on the adsorption plot (Benhima et al. 2008). At this stage, the sorbent is said to have attained its operational maximum adsorption capacity.

When the experimental data were treated with the Langmuir model of adsorption, the linear regression was *R* ^2^ = 0.775, but when treated with the Freundlich model, it had a linear regression of *R* ^2^ = 0.450 favouring the Langmuir model of adsorption (Freundlich 1906; Langmuir 1918). This implies that the interaction was chemisorption (Deng et al. 2003). The calculated adsorption capacity was 2.068 mg g^−1^ which agrees well with the value obtained graphically of 2.00 mg g^−1^.

## Conclusions

This study successfully developed a spectroscopic method for the determination of polyDADMAC at trace levels by exploiting the epoxidation of double bonds within its chemical structure. The epoxidation process was confirmed with FTIR analysis and supported by ^1^H NMR. This process allowed the UV–Vis inactive compounds, such as polyDADMAC, to be successfully be determined by this UV–Vis technique. The calibration curve obtained showed a linear relationship with a correlation coefficient (*R* ^2^) of 0.993 giving a molar adsorptivity of 1.929 g^−1^ cm^−1^. The detection limit was found to be 2.1 × 10^−4^ μg L^−1^ limit of detection of 2.2 × 10^−4^ μg L^−1^, indicating therefore that it is suitable for trace analysis. The advantage of the developed method is that the UV–Vis technique is simple and does not require specialised skills. Furthermore, the technique is fast, inexpensive and satisfactory for routine analysis of polyDADMAC residue at trace levels in treated waters.
